# Can mergers-in-progress be unmerged in speech accommodation?

**DOI:** 10.3389/fpsyg.2013.00653

**Published:** 2013-09-24

**Authors:** Molly Babel, Michael McAuliffe, Graham Haber

**Affiliations:** Department of Linguistics, University of British Columbia, VancouverBC, Canada

**Keywords:** phonetic imitation, accommodation, mergers, dialects of English, phonetics

## Abstract

This study examines spontaneous phonetic accommodation of a dialect with distinct categories by speakers who are in the process of merging those categories. We focus on the merger of the NEAR and SQUARE lexical sets in New Zealand English, presenting New Zealand participants with an unmerged speaker of Australian English. Mergers-in-progress are a uniquely interesting sound change as they showcase the asymmetry between speech perception and production. Yet, we examine mergers using spontaneous phonetic imitation, which is phenomenon that is necessarily a behavior where perceptual input influences speech production. Phonetic imitation is quantified by a perceptual measure and an acoustic calculation of mergedness using a Pillai-Bartlett trace. The results from both analyses indicate spontaneous phonetic imitation is moderated by extra-linguistic factors such as the valence of assigned conditions and social bias. We also find evidence for a decrease in the degree of mergedness in post-exposure productions. Taken together, our results suggest that under the appropriate conditions New Zealanders phonetically accommodate to Australian English and that in the process of speech imitation, mergers-in-progress can, but do not consistently, become less merged.

## Introduction

Speech is a highly variable behavior. This variability is conditioned in part by the multiple degrees of freedom involved in the highly complex act of producing speech: an individual will never produce a word exactly the same way twice, though perceptual constancy ensures that the perception of variable productions remains relatively constant. This within-speaker variability along with the physiological differences between speakers and the co-mingling of speakers from multiple dialect and language backgrounds in urban settings, attest to the great phonetic variability in spoken language. Classic papers like Peterson and Barney (1952) and Hillenbrand et al. (1995) showcase the massive amount of overlap of phonetic categories seen when describing vowel systems even within a single speech variety. Despite this significant overlap and constantly variable signal, speakers and listeners successfully map an utterance onto linguistically meaningful categories. Listeners' task of making sense of the signal is made even more challenging by the social and indexical factors which also condition variation within and across speakers (e.g., Labov, 1963).

Within the synchronic pool of variation in which listeners are immersed, there is evidence for sound changes in progress. A sound change which presents a particularly difficult challenge for the listener is the merger. Mergers are a type of sound change that involve the elimination of a contrast between two formerly distinct phonemic distributions. Take, for example, the 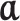/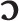 of the COT/CAUGHT merger found in several dialects of the United States and Canada. While it was previously the case that words like *cot* and *caught* or *stock* and *stalk* were pronounced with different vowels—e.g., /k^h^
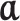
t/ and / /k^h^
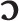
t/ or /st
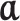
k/ and /st
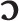
k/, many dialects of North American English have merged these sets of words such that there is no longer a reliable difference in their pronunciation. Some dialects are currently in the process of merging these lexical sets, while others still retain the contrast. Many mergers completely finish; we can consider *nose* and *knows* as such an example. *Nose* and *knows* were once pronounced as /nu:z/ and /nΛuz/ in Middle English, and have since completely merged to a homophonous pronunciation in nearly all varieties of modern English, pronounced as /$\text{n}\overset{⌢}{\text{o}℧}\text{z}$/ in most varieties of North American English.

**Figure 1:**
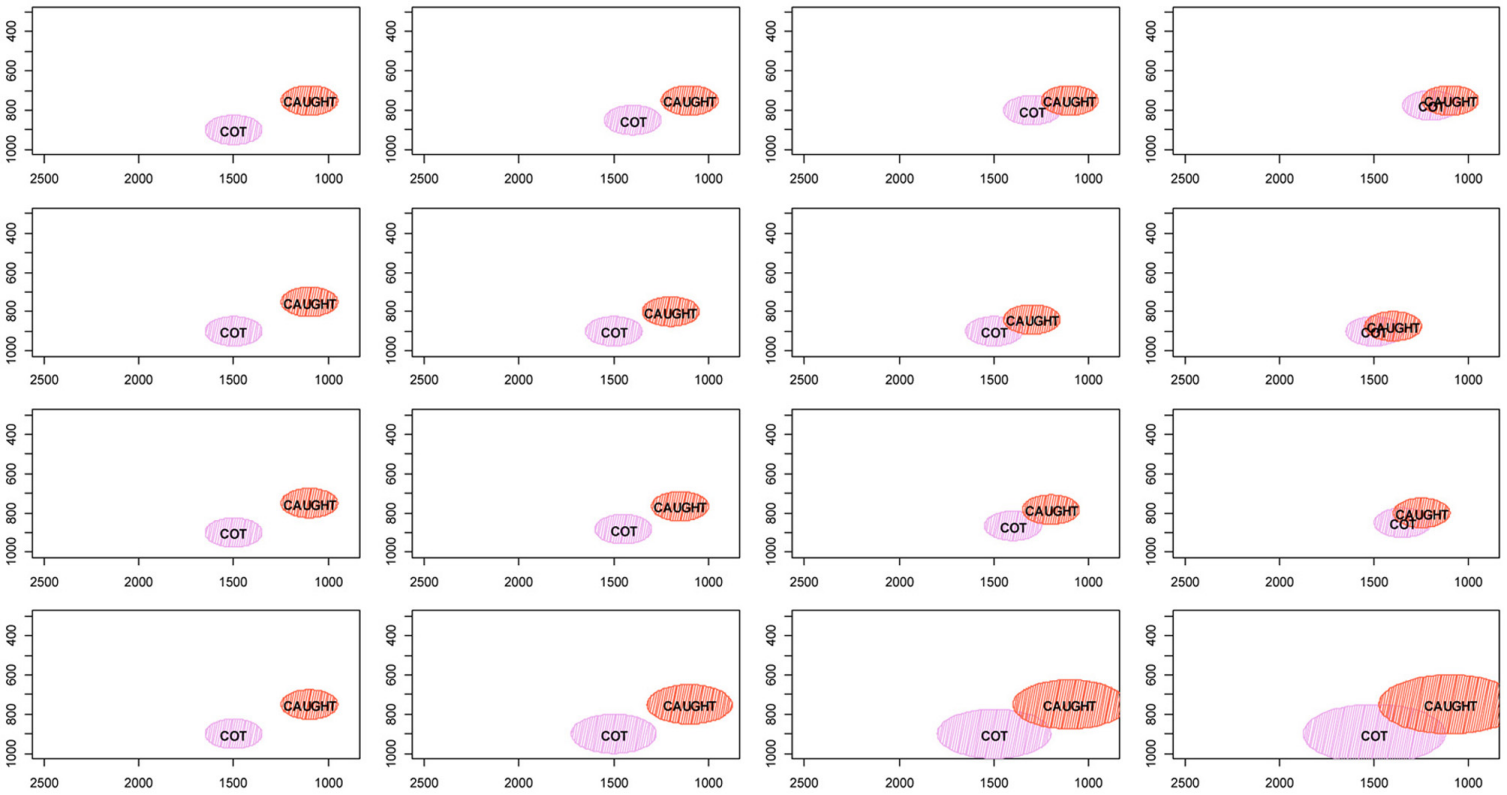
**Schema illustrating the four ways in which mergers can be achieved using the CAUGHT-COT merger as an example.** The shifting of one category into the territory of another is shown in the first and second rows. The third row illustrates merger by symmetrical approximation. The fourth row demonstrates merger by expansion.

The merger of two phonetic categories can be achieved by several means, as illustrated in Figure 1, where each row represents a path toward merging and the columns represent different points in time with the leftmost column showing the initial state of the system before the merger and the rightmost column the merger realized. The CAUGHT/COT merger serves as the example in this figure. The first three rows present different types of mergers by approximation (Trudgill and Foxcroft, 1978). The first and second rows illustrate mergers caused by the phonetic drift of one of the two categories. In the first row COT is lowering in the first and second resonant frequencies of the vocal tract (henceforth F1 and F2, respectively), and thereby merging with the CAUGHT distribution. The second row shows the opposite pattern: CAUGHT is raising in F1 and F2 and merging with the distribution of the COT category. The third row illustrates a symmetrical merger by approximation, where the categories' distributions drift together into a novel acoustic-phonetic space. Expansion (Labov, 1994; pp. 321–323) is shown in the fourth row of the figure; merger by expansion is accomplished by the distributions of both sounds growing to the point of category merger.

Mergers provide a poignant example of the separation between perception and production, and a clear case of how an individual's language system is not contained and constrained by their own productions. An exploration of this separation between perception and production involves an assessment of whether speakers naturally produce the contrast, and whether they are able to perceive the contrast in the productions of themselves and others. Here, perceiving the contrast means being able to correctly identify the intended lexical items. DeCamp (1953) originally documented how, as the COT/CAUGHT merger was progressing through the speech patterns in San Francisco, individuals could fall under one of four types: there were individuals who were (1) naturally unmerged in production and unmerged in perception; (2) merged in production and merged in perception; (3) merged in their own productions, yet able to perceive the contrast in the speech of others; and (4) unmerged in their own productions, but unable to perceive the contrast in their own productions or those of other unmerged speakers. This final case of those who reliably produce two separate phonetic categories, but are unable to perceive the contrast, was noted as a curious finding and has since been replicated by Labov et al. (1991). In short, in speech communities where a merger is in progress, individuals' systems can vary in whether they produce and/or perceive the merger.

Mergers therefore showcase the lack of isomorphism in an individual's speech perception and production systems. As users of language we can perceive many more varieties of speech than we can ever produce: females and males understand each other despite large differences in production; a speaker of American English can understand Southern Standard British English, yet not be able to produce sounds in the same way; and a toddler can understand an adult's spoken language despite not having acquired the target productions of the variety being acquired. Uniformity in production is not necessary in order to achieve perceptual parsimony.

While mergers-in-progress showcase a cognitive division between perception and production, spontaneous phonetic imitation illustrates the connection between the two faculties. Spontaneous phonetic imitation is the unconscious process by which exposure to a speech stimulus causes a listener-turned-talker to display characteristics of the stimulus in their own productions (Goldinger, 1998; Namy et al., 2002; Shockley et al., 2004; Babel, 2010, 2012; Miller et al., 2010; Nielsen, 2011; Babel and Bulatov, 2012). In spontaneous phonetic imitation the perceptual input has a clear and immediate impact on the production output. Of course, not all participants imitate, nor do they imitate to the same extent. In fact, calling the phenomenon *imitation* is somewhat of a misnomer. As noted in the opening paragraph, an individual can never perfectly replicate her own production (Vallabha and Tuller, 2004), let alone exactly match or imitate that of another speaker with a different vocal tract. The term *spontaneous phonetic imitation* has been used in the literature since Goldinger (1998) as a way of referring to the automatic and subconscious means by which low-level phonetic details are picked up through simple exposure (but see Mitterer and Ernestus, 2008). A long line of literature under Communication Accommodation Theory (CAT; e.g., Giles, 1973; Giles et al., 1991) has similarly examined how language users converge in interaction, but the crucial difference between CAT and research using the term spontaneous phonetic imitation is that CAT sees the phenomenon as one of speakers, consciously or not, converging to decrease social distance and facilitate interaction. This contrasts to the perspective that convergent behavior is the consequence of the organization of linguistic systems (Goldinger, 1998; Pickering and Garrod, 2004).

Indeed, episodic models of speech are often used to model the results of phonetic imitation as they allow for perceptual input to be incorporated into production output. Pierrehumbert's (2002) neo-generative model of speech perception and production posits abstract phonological generalizations and representations which are associated with parametric multidimensional auditory-phonetic distributions. These distributions are activated at a phonetic implementation stage in production, allowing previously experienced tokens to influence the output through the perception-production loop. Pierrehumbert's model, however, lacks the attention-weighting mechanism in Johnson (1997) which builds upon Nosofsky (1986). Attention-weighting mechanisms are necessary to capture the fact that perception is context specific, and that not all information is attended to or treated in a homogenous way. Such attention-weighting is necessary to account for the fact that phonetic imitation, like other forms of behavioral alignment (Dijksterhuis and Bargh, 2001), is facilitated by social factors such as liking (Babel, 2010, 2012). This truly echoes earlier and ongoing work which falls under CAT. Moreover, CAT documents and theorizes the response opposed to that of accommodation namely, that of divergence where interacting individuals come to be less similar linguistically as a means of increasing social distance. Additional evidence from perception experiments on listeners' control over speaker normalization processes provides further evidence for the need for an attention-weighting or a listener dependent strategic control mechanism in an episodic language system (Magnuson and Nusbaum, 2007; Barreda, 2012).

Trudgill (2008) has suggested that the real life context for phonetic imitation is new dialect acquisition. While variable, adults generally acquire aspects of a new dialect when they move to other dialect regions (Trudgill, 1986; Munro et al., 1999; Evans and Iverson, 2007). Using an experimental paradigm, Delvaux and Soquet (2007) show how exposing Mons Belgian French speakers to a different regional dialect, Liège, induces shifts toward the Liège dialect in a sentence production task. In a particularly well-documented case, Harrington and colleagues described Queen Elizabeth II's acquisition of more Estuary English-influenced speech patterns over the decades (Harrington et al., 2000a,b; Harrington, 2006, 2007). These changes speak to the dynamism of the phonetic system, and how with age, the system does not fossilize completely. While adults' abilities to acquire new phonetic systems may attenuate with age, it does not disappear completely. Moreover, the newly acquired sounds seem to function as additions to a speaker's phonetic repertoire, not categorical replacements (Howell et al., 2006).

In the context of dialect acquisition, mergers prove to be challenging to learn in that, in fact, once merged categories have been acquired, it is difficult to unlearn such patterns. Returning to the homophony between *nose* and *knows* described earlier, Trudgill (1981) studied a group of children who were native speakers of a dialect where *nose* and *knows* were homophonous. These children then moved to Norwich where the local variety of English had maintained the Middle English contrast, and pronounced these words differently. The merged children were not able to fully acquire the unmerged system. A similar pattern has been found for adults. Evans and Iverson (2007) tracked a group of young adults from Ashby de la Zouch, Leicestershire, a small Midlands town, where the local accent is a variety of northern English. These students moved to different parts of England for university where they were exposed to Standard Southern British English (SSBE). The researchers charted the students' adoption of SSBE features during their time in university. In their Ashby dialect *could* and *cud* are both produced with /℧/, while in SSBE *could* is pronounced with /℧/ and *cud* with /Λ/. They found that both *could* and *cud* were shifting away from /℧/ and toward /Λ/. That is, there was no unmerging of the categories, rather the entire merged category was shifting toward the SSBE vowel in *cud*.

The merger of interest in this paper is the merger of the NEAR and SQUARE lexical sets in New Zealand English. The lexical sets for NEAR and SQUARE have been merging since at least the 1970s (Maclagan and Gordon, 1996; Gordon and Maclagan, 2001). Typically, this merger-in-progress involves raising of the SQUARE diphthong such that in merged speakers, the vowel is realized as [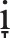
ə] (Hay et al., 2006) and in doing so, approximates the NEAR vowel, making this an asymmetrical merger of approximation.

How flexible are speakers' representations of mergers-in-progress? Warren et al. (2007) showed that New Zealand listeners have high accuracy rates when identifying NEAR/SQUARE words produced by an unmerged speaker, and that as individuals' degree of mergedness increases, their error rates in word identification do as well. They suggest that this relationship between perception and production is not causal, however; an individual who is more merged is likely to interact with others who are also more merged, reducing his or her experience with unmerged talkers and thereby increasing the perceptual difficulty of the task. Listeners' abilities to accurately identify NEAR/SQUARE words is also tied to whether they *expect* a voice to be merged or unmerged. Using a matched-guise face-priming paradigm, Hay et al. (2006) show that listeners have higher error rates when an unmerged voice is co-presented with a picture suggesting youth or lower socioeconomic status, populations which are generally more advanced with respect to the merger-in-progress. Listeners adapt their perceptual expectations to the social characteristics of a speaker or apparent-speaker.

Do speakers of New Zealand English exhibit analogous flexibility in production? To explore speakers' flexibility in the production of the NEAR/SQUARE merger, we used an auditory naming task, which is typical of the spontaneous phonetic imitation paradigm (Goldinger, 1998). We presented New Zealand participants with a speaker of Australian English. Australian English is not undergoing this merger, and the model talker's productions of these diphthongs were unmerged with NEAR as /iə/ and SQUARE as /eə/. Research on linguistic and behavioral accommodation suggests that imitative behavior is not simply about exposure, but is a process facilitated by social preferences (e.g., Babel, 2010, 2012) and is used to establish social cohesion (e.g., Carpenter et al., 2013). To this end we included two manipulations in the experimental design to probe how social distance affects phonetic imitation with the NEAR/SQUARE merger. In a positive valence condition (Positive Condition), participants were presented with a text which described the Australian talker's positive feelings toward New Zealand. Those in a negative valence condition (Negative Condition) were presented with a text which described the Australian model talker's negative attitude toward New Zealand. To measure individual preferences for New Zealand and Australia participants completed an Implicit Association Task (IAT; Greenwald et al., 1998) to determine their biases toward New Zealand and Australia.

## Auditory naming task

### Participants

Forty-two participants (females = 34, males = 8) from the Victoria University of Wellington community completed an auditory naming task. Male and female participants were evenly assigned to the two conditions, which are described below. The task took approximately 30 min and participants were compensated with a $10 book voucher.

### Materials

The auditory stimuli were single word productions from a 32 year old male talker who was born and raised in Melbourne, Australia. At the time of the recording the talker was living in Berkeley, California and was recruited through personal contacts. A subset of the single words contained diphthongs involved in the NEAR/SQUARE merger in New Zealand English. The diphthongal stimuli list was taken from that used by Hay et al. (2006). The list of NEAR/SQUARE words analyzed in this paper is shown in Table 1. The pair *mere*/*mare* was included in the list, but due to a spelling error rendering *mere* as *meer*, this pair was not analyzed. Participants were also additionally presented with monophthongal stimuli taken from the lexical sets KIT, DRESS, TRAP, BARN, STRUT, and THOUGHT. Only the results from the diphthongs are presented in this paper.

**Table 1:** **Diphthong minimal pairs under investigation in the current study**.

**NEAR**	**SQUARE**
Ear	Air
Beer	Bare
Dear	Dare
Fear	Fare
Hear	Hair
Peer	Pair
Really	Rarely
Sheer	Share
Spear	Spare

### Procedure

Participants were seated at a PC laptop and the experiment was presented using E-Prime 2.0 Experimental Software (Psychology Software Tools, Pittsburgh, PA). Auditory stimuli were presented over AKG K270 headphones. Audio-recording was done directly in E-prime using an M-Audio USB audio device with a head-mounted AKG C520 microphone positioned three inches from the participant's mouth. The task was designed as follows: Participants were randomly presented with hVd words (hid, had, head, etc.) which they were to read aloud. Participants then were presented with the target word list which they were also asked to produce aloud; these productions serve as the baseline or pre-task productions. The order of words in each list was fully randomized for each participant. The following block was the shadowing block where participants were exposed to the target word productions from the Australian model talker over headphones. Words were randomly presented twice across two test blocks, creating blocks referred to as Shadowed 1 and Shadowed 2. There was no break between blocks and each word was presented once in each block. Word order was fully randomized in each block. Participants' instructions for this part of the task were to identify the word heard by saying it out loud. Participants then did a post-task reading of the wordlist; this block was identical to the pre-task block, except that the words were presented in a different random order. Finally, participants read the hVd words again. Comparing baseline, shadowed, and post-task productions, we can examine how New Zealand participants modify their productions as a result of exposure to the Australian model talker.

Participants were assigned to one of two valence conditions. In the Positive Condition, participants were presented with the following text which was intended to make them view the talker and Australia as a whole in a positive light:

The Australian talker you are about to hear was actually born in Auckland. At a young age, however, he and his parents moved to Melbourne where he has lived since. His grandparents and the rest of his extended family still live in New Zealand, so he visits frequently. In fact, he is currently looking for employment in New Zealand so that his children may live closer to their great-grandparents.

The second condition was a Negative Condition. The purpose of this condition was to inspire negative feelings toward the talker and Australia.

The Australian talker you are about to hear was born in Sydney. Like many Australians, he has strong negative opinions of New Zealand. For one, he thinks that New Zealanders are rather stupid and that they lack culture. In addition, he finds the entire population backwards and naïve. In his mind, New Zealand is provincial and has a horrid cricket team. He never intends to visit New Zealand because of these views.

In both Positive and Negative conditions participants were exposed to a screen which displayed the assigned text immediately before beginning the shadowing portion of the task. After reading the Positive or Negative text participants pressed a button that took them to the test-block.

Upon completion of the speech production task participants completed an Implicit Association Task (IAT; Greenwald et al., 1998). There are five blocks in this task. The first block is target-concept discrimination. The targets: Australia and New Zealand are presented on opposite sides of the monitor. A combination of Australian or New Zealand concepts—famous individuals, maps, and images (flags, native scenery, sports emblems, etc.)—were then randomly presented (e.g., an image of a kangaroo or an image of a kiwi) in the middle of the screen. A participant's task is to categorize the concepts as Australia or New Zealand as quickly as possible. The second block is associated attribute discrimination. The attributes good and bad are presented on opposite sides of the monitor in place of Australia and New Zealand. Attribute words are presented randomly (e.g., rainbow or cancer) in the middle of the screen. Participants categorize the words as semantically good or bad words. The third block is a combined test block. Labels for the concept categories (Australia vs. New Zealand) and word (good vs. bad) attributes are presented at the top corners of the screen. In the center, either a concept or a word are randomly presented and must be categorized. Participants are instructed to ignore the target-concept when categorizing words and ignore the attributes when categorizing concepts. Concepts that are words are presented in all capital letters and words are presented in all lowercase letters to facilitate the process. Block 4 is just like Block 1, except that the labels Australia and New Zealand are presented on different sides of the screen (so, if Australia was on the right-side of the screen in Block 1, it was on the left side in Block 4). Participants then categorized concepts as Australia or New Zealand as they did in Block 1.

Block 5 is the reversed combined task; the reversed order of the target-concepts (Australia and New Zealand) are matched up with the original order of good and bad such that if Australia was originally presented above good and New Zealand with bad, this pattern is reversed and Australia is presented with bad and New Zealand with good. The experiment was counterbalanced so that half of the participants were initially exposed to Australia paired with good and New Zealand paired with bad while the other half were first presented with Australia paired with bad and New Zealand paired with good.

Participants logged responses using assigned buttons on a computer keyboard. Responses were collected automatically using E-prime. Participants' scores were calculated using the updated methods described in Greenwald et al. (2003).

The institutional ethical review boards at the University of California, Berkeley and Victoria University at Wellington approved the speech production experiment. Informed consent was obtained from all research participants.

## Quantifying imitation

We quantify imitation in two ways. First, we measure phonetic accommodation using an AXB perceptual similarity task. We then acoustically quantify the degree of mergedness of the diphthongs using Pillai-Bartlett traces.

### Perceptual judgments

#### Methods

***Participants.*** One hundred and sixty-two self-identified native speakers of North American English from the University of British Columbia community participated as listeners. Listeners reported no speech, language, or hearing disorders and were compensated $10CAN for their time.

***Materials.*** Participants' baseline and shadowed productions and the model talkers' productions were used as stimuli in this task. Forty-one of the original 42 shadowers were used in this task. One was removed because the majority of her productions were initiated toward the end of the pre-set recording time and the ends of her productions were, therefore, cut off. The baseline, shadowed productions, and post-task productions of the remaining 41 shadowers were used along with the tokens from the Australian model talker.

***Procedure.*** Listeners were seated at a computer workstation and presented with auditory stimuli over AKG K240 headphones. Stimuli were presented using E-Prime experimental software (Schneider et al., 2007). The basic procedure was an AXB similarity judgment. Each trial consisted of three sound files separated by a 300 ms ISI. The middle token (X) was always a token from the model Australian, and the first (A) and third (B) tokens were baseline, shadowed, or post-task productions from a single participant. A baseline token was used in each trial such that across the Shadowed 1, Shadowed 2, and Post-task productions, the comparison was always relative to a participant's baseline production. Each trial consisted of a single lexical item, and each potential trial was played twice to counterbalance the order of the baseline and the post-exposure token (shadowed or post-task). Within each shadower, the order of presentation was fully randomized. Due to the large number of tokens, each listener was randomly presented with four shadowers, two from the Positive Condition and two from the Negative Condition; each shadower was assigned to an average of 15 listeners. Listeners were offered short breaks between shadowers.

Listeners' task was to determine which participant production sounded more like the model talker. If listeners consistently select a Shadowed or Post-task token as more similar that is taken as evidence for imitation. If listeners consistently select the baseline token as more similar to the model's production, then that is taken as evidence of divergence; that is, the participant sounded more like the model during baseline productions and sounded *less like* the model during or after exposure. If listeners choose baseline and post-exposure tokens with equal probability, it suggests that the shadower did not modify her or his speech as a result of exposure to the model.

The institutional ethical review board at the University of British Columbia approved the speech perception task. Informed consent was obtained from all research participants.

#### Results and analysis

To eliminate inattentive responses, those with response times greater than two standard deviations from the mean were eliminated. This resulted in the removal of 5.3% of the data set. With the remaining data, a mixed effects logistic regression model predicting the proportion of shadowed judgments selected by listeners as more similar-sounding to the model was fit with Block (Shadowed 1, Shadowed 2, Post-task), diphthong Category (NEAR, SQUARE), and Condition (Positive, Negative) as predictor variables. In each trial, a shadowers' baseline production was presented with a token from a later block such that in the statistical analysis Block compares tokens from Shadowed 1, Shadowed 2, and the Post-task to the Baseline tokens. To simplify the analysis we have omitted IAT scores and analyze their contribution to participants' behavior in a separate analysis below. All categorical variables used treatment coding. Listener, Shadower, and Word were entered as random effects. There were by-Listener random slopes for diphthong Category and Condition; diphthong Category was also a by-Shadower random slope; and Condition was a by-Word random slope. This was the maximal random effects structure which still converged for the model; this method is used following the recommendations of Barr et al. (2013). The reference level for Condition was the Positive Condition, and the reference level for diphthong Category was the SQUARE lexical set. Block was the only factor with more than two levels; the first shadowing block (Shadowed 1) was the reference level in the first analysis, allowing comparisons between Shadowed 1—Shadowed 2 and Shadowed 1—Post-task (for a discussion on issues that arise when using mixed effects models with multi-level factors see Clopper, 2013). The intercept of this model was significant and went in a positive direction, indicating phonetic imitation for the model as a whole (β = 0.22, *SE* = 0.065, *p* < 0.001). A second model was also run where the reference level for Block was set to the Post-task, allowing for the comparisons Post-task-Shadowed 1 and Post-task-Shadowed 2; this was the only difference between the two models. The intercept for the model with the Post-task as the baseline reference level for the Block variable was not significant (β = 0.04, *SE* = 0.065, *p* = 0.50). We present the significant results of the models along with figures illustrating the results in the paragraphs that follow.

**Figure 2:**
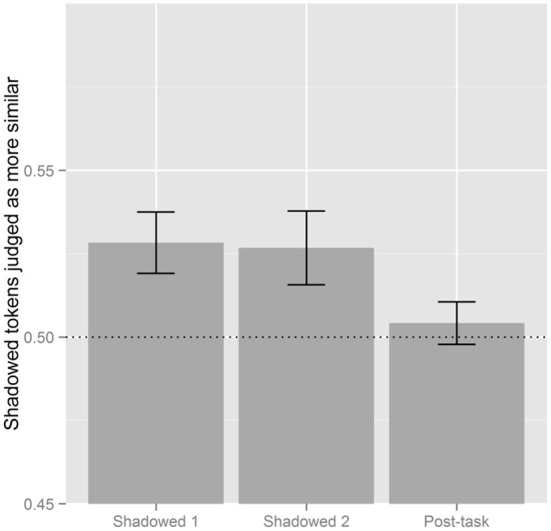
**Visual summary of the results by Block.** Proportions above 0.5 indicate that Shadowed or Post-task tokens are more judged to be more similar to the model, while values below 0.5 means baseline tokens were more likely to be judged as similar to the model's production.

With Shadowed 1 as the reference level there was an effect of Post-task (β = −0.177, *SE* = 0.039, *p* < 0.001), and the same effect of Shadowed 1 was found with Post-task as the reference level (β = 0.177, *SE* = 0.039, *p* < 0.001). When Post-task was the reference level there was also a significant effect of Shadowed 2 (β = 0.132, *SE* = 0.039, *p* < 0.001). Figure 2 illustrates these effects; listeners perceived less accommodation in the Post-task than in Shadowed 1 and Shadowed 2, and there is no difference between the two shadowing blocks. In this figure, and others like it which follow, the vertical axis reports the proportion of shadowed and post-task tokens which were judged as more similar to the model than a shadower's baseline productions. Proportions above 0.5 indicate that post-exposure tokens were judged as more similar while values below 0.5 suggest that shadowers' baseline tokens sound more similar.

**Figure 3:**
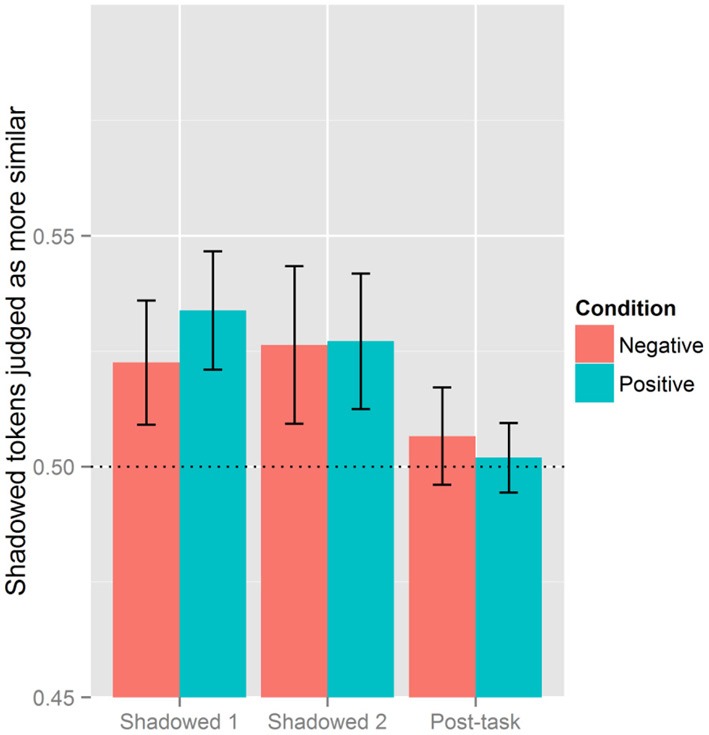
**Effect of block and condition.** The vertical axis is the proportion of post-exposure tokens judged as more similar to the model. Proportions above 0.5 indicate that Shadowed or Post-task tokens are more judged to be more similar to the model, while values below 0.5 means baseline tokens were more likely to be judged as similar to the model's production. The line at 0.5 serves to indicate chance levels.

With Shadowed 1 as the reference level there was an interaction with Block (Shadowed 2) × Condition (Negative) (β = 0.131, *SE* = 0.055, *p* < 0.05) and Block (Post-task) × Condition (Negative) (β = 0.151, *SE* = 0.055, *p* < 0.01). Block (Shadowed 1) and Condition (Negative) went on to interact when the Post-task was the reference level (β = −0.151, *SE* = 0.055, *p* < 0.01). This is shown in Figure 3 illustrating how listeners perceived the most accommodation in Shadowed 1 for those in the Positive Condition; the difference across conditions attenuates in the second shadowing block. In the Post-task, listeners perceive the lowest levels of accommodation in the Positive Condition.

**Figure 4:**
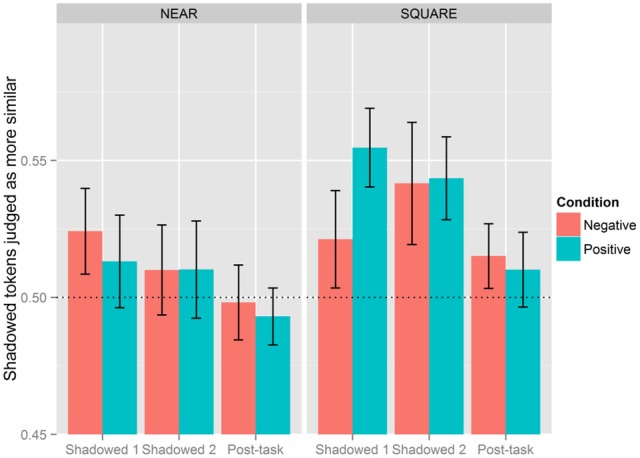
**Effect of condition, block, and category.** The vertical axis is the proportion of post-exposure tokens judged as more similar to the model. Proportions above 0.5 indicate that Shadowed or Post-task tokens are more judged to be more similar to the model, while values below 0.5 means baseline tokens were more likely to be judged as similar to the model's production. The line at 0.5 serves to indicate chance levels.

Block (Shadowed 2), Category (NEAR), and Condition (Negative) went on to interact as an effect with Shadowed 1 as the reference level (β = −0.159, *SE* = 0.078, *p* < 0.05), in addition to an interaction involving Block (Post-task), Category (NEAR), and Condition (Negative) (β = −0.157, *SE* = 0.078, *p* < 0.05). The inverse of this three-way interaction also surfaced when the Post-task was the reference level [Block (Shadowed 1) × Category (NEAR) × Condition (Negative): β = 0.157, *SE* = 0.078, *p* < 0.05].^1^ Figure 4 presents listeners' judgments of accommodation by Block, Condition, and diphthong Category. As is clear from this figure, listeners generally perceived imitation more with the SQUARE words than NEAR words, but this was subject to additional effects of Condition and Block. While listeners generally judged there to be more accommodation with SQUARE words, this pattern does not hold for Shadowed 1 in the Negative Condition; there, shadowers imitated SQUARE to the same extent as NEAR. Shadowers in the Negative Condition, however, increased their imitative behavior of SQUARE to the same level as those in the Positive Condition in Shadowed 2.

**Figure 5:**
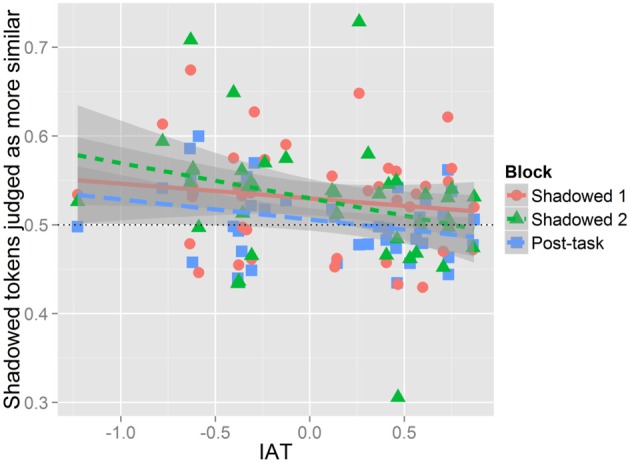
**IAT by listeners' judgments of imitation.** The x-axis presents IAT scores, and the y-axis is the proportion of tokens judged as more similar to the model for each block. Each dot on the figure represents the mean proportion of perceived convergence in a block for a single participant. Data from Shadowed 1 is in red, Shadowed 2 is green, and Post-task is blue. Negative IAT scores indicate a positive bias toward Australia, and positive IAT scores indicate a positive bias toward New Zealand. Proportions above 0.5 indicate that Shadowed or Post-task tokens are more judged to be more similar to the model, while values below 0.5 means baseline tokens were more likely to be judged as similar to the model's production.

To assess how IAT scores predict shadowers' accommodative behavior the averaged proportions of listeners' judgments of shadowed and post-task productions for each shadower for each block were compared to each shadowers' score on the IAT. Given how the IAT was scored, negative IAT values indicate a pro-Australian bias while positive IAT values indicate a pro-New Zealand bias. Assessing the existence and nature of the effect IAT has on perceived imitation, we found a significant negative correlation [*t*_(121)_ = −2.76, *r* = −0.24, *p* < 0.01]; this suggests that shadowers who were pro-Australian were more likely to spontaneously imitate the Australian model regardless of their assignment to the Positive or Negative Condition. This pattern is shown in Figure 5 where the aggregate data and regression lines are plotted for each block; data from Shadowed 1 is shown in red circles, Shadowed 2 in green triangles, and the Post-task in blue SQUARES. Presenting the data in this format allows for the observation that while there is a negative slope for each block, IAT had a reduced effect on Shadowed 1. The slope is steeper in Shadowed 2, illustrating there is more variation in performance across shadowers who differ in IAT scores.

#### Discussion

The perceptual judgments of phonetic imitation resulted in a complex set of results. Phonetic imitation was affected by individuals' task block, valence condition assignment, and diphthong category. Shadowers imitated more in the shadowing blocks than they did in the Post-task. While there was no difference overall across the two shadowing blocks, listeners perceived more accommodation in the voices from those who had been assigned to the Positive Condition than the Negative Condition in Shadowed 1, and this pattern dissipated by Shadowed 2. While shadowers generally accommodated in the second shadowing block as well, there was more variability across shadowers in Shadowed 2; such a finding suggests that initial cross-dialect phonetic imitation may be facilitated by exposure to novel stimuli. IAT scores were correlated with listeners' judgments of phonetic imitation as well. Overall, the more positively shadowers viewed Australia, the more they were judged as converging.

The lexical set of each item also affected the extent of phonetic imitation. Listeners judged more imitation in the SQUARE words than in the NEAR words. For those assigned to the Positive Condition, SQUARE was imitated more than NEAR in both shadowing blocks, but this pattern did not persist into the Post-task. For the Negative Condition, SQUARE was imitated more than NEAR in Shadowed 2, but this was not the case for Shadowed 1 or the Post-task. Given that in the NEAR/SQUARE merger, the SQUARE category is generally moving toward the NEAR category, we would expect that speakers of New Zealand English would have a larger phonetic repertoire for SQUARE words, allowing for more phonetic imitation for this category (e.g., Babel, 2010, 2012; Kim et al., 2011). Such a conclusion would be hasty, however, without acoustically analyzing the degree of merger for participants across blocks. The perceptual measure of accommodation has indicated that participants indeed accommodated to varying degrees with the NEAR/SQUARE words in this task. The following section addresses the extent to which the merger is attenuated in phonetic imitation.

### Acoustic analysis of diphthongal merger

A principal goal of this study is to examine whether New Zealand speakers can change the degree of their NEAR/SQUARE merger when exposed to an Australian speaker. To achieve this goal, we first conducted an acoustic analysis for each vowel, and then quantified the degree of merger.

Previous study of the NEAR/SQUARE merger has used Bark-scaled F1 and F2 at the point of highest F2 during the first element of the diphthong (Hay et al., 2006). This approach only considers a single point in a dynamic trajectory. One way of capturing dynamic information is to perform a discrete cosine transform on time series data from the vowel. Discrete cosine transforms of a given formant returns three primary coefficients which can be used for further analysis. The first coefficient corresponds to the overall position of the formant in the vowel space, the second corresponds to the slope of the formant over time, and the third corresponds to the curvature of the slope. Essentially, each coefficient captures deviation from the previous coefficient, and the first captures deviation from zero. Discrete cosine transforms of the second formant have sought to capture variation from coarticulatory effects (Kleber et al., 2012), where greater Euclidean distance from a neutral production indicates a trajectory with more influence from surrounding consonants.

Euclidean distance is one possible measure of mergedness. As pointed out by Hay et al. (2006), Euclidean distance, however, does not take into account distributional information. For instance, if two categories vary widely in their productions, their means can be relatively far apart while their distributions largely overlap. In such a scenario, Euclidean distance measures would inaccurately report a large separation, regardless of their overlapping distributions. To this end, Hay et al. (2006) use Pillai-Bartlett scores to measure overlap between NEAR and SQUARE categories in F1 and F2 dimensions. The Pillai score is a summary statistic of multivariate analyses of variance (MANOVA), the scores of which describe the separability of two distributions as well as variation within each distribution. Scores close to 0 correspond to overlapping or merged categories and scores close to 1 corresponding to distinct categories with no between-category variation; a value of less than 1 can still indicate separate categories with some amount of overlap between categories.

#### Methods

The approach for determining the degree of a given speaker's NEAR/SQUARE merger used here combines these two previous approaches of discrete cosine transforms and Pillai scores. Discrete cosine transforms are performed on participants' productions, and Pillai scores are calculated based on the first coefficient of F1 and F2 transforms. The first coefficient of the transform is similar to the point measure in that it is a location in Bark-scaled formant space, but it also contains dynamic information not present in point measures. A MANOVA of the coefficients by diphthong category was performed for each block for each subject, giving four Pillai scores for each shadower over the course of the experiment (one for their Baseline productions, Shadowed 1 productions, Shadowed 2 productions, and Post-task productions).

Acoustic analysis of vowel productions was performed using FAVE (Rosenfelder et al., 2011). FAVE builds on the formant prediction algorithm implemented in Evanini (2009), which correlates well with manual formant measurements from sociolinguistic data. For a given vowel, a 12^th^ order linear predictive coding analysis is performed and the poles and bandwidths are extracted. Each possible F1 and F2 combination of the six extracted poles is evaluated to find the best combination. The best combination is the one that minimizes the Mahalanobis distance to the vowel's category means in four parameters (F1, F2, and their respective bandwidths). The FAVE analysis used here performs two passes over the data. The first pass uses vowel category means from the Atlas of North American English (Labov et al., 2005), and the second pass uses vowel category means generated from each speaker. While FAVE is most suitable for North American varieties of English, using the speakers' own means for re-measurement allows for accurate measurement. FAVE outputs five formant measurements over the middle 60% of the vowel (20%, 35%, 50%, 65% and 80%), and these measurements were the basis of the discrete cosine transform (DCT).

Following acoustic analysis, Pillai scores for each speaker in each production block were calculated. Each production block had 18 NEAR/SQUARE words. A multivariate analysis of variance (MANOVA) was performed with the first coefficient of the F1 DCT and of the F2 DCT as the dependent variables and diphthong Category as the sole independent variable. From this MANOVA, the Pillai score of that speaker's production block was extracted, and ranged from 0 (completely merged) to 1 (completely distinct). Following the within-subject, by-block MANOVAs, a by-subject linear mixed-effects model was constructed with Block (Baseline, Shadowed 1, Shadowed 2, and Post-task), Condition (Positive and Negative), and their interactions as fixed effects. As Block is the only factor repeated across subjects, it was the only random slope specified in the model, corresponding to the maximal random effect structure for this analysis. Because the Pillai score is a distributional measure, a by-subject analysis is the only one possible. There may well be item effects present in this study, but a different analysis of the degree of merger would be necessary to investigate such effects.

#### Results

**Figure 6:**
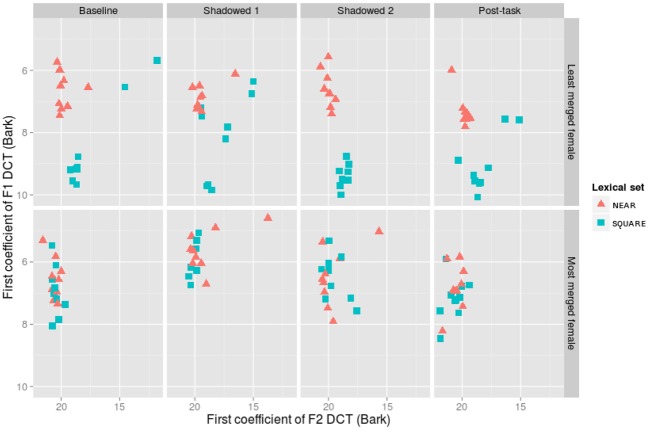
**The first coefficients for the discrete cosine transform (DCT) of F1 and F2 for the overall least merged (row 1) and most merged (row 2) female participants**.

**Figure 7:**
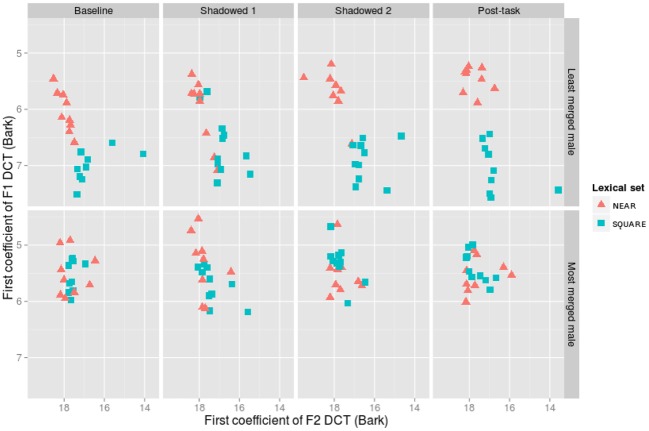
**The first coefficients for the discrete cosine transform (DCT) of F1 and F2 for the overall least merged (row 1) and most merged (row 2) male participants**.

***Overview of Pillai scores.*** In Figures 6, 7, vowel plots using the first coefficient of the discrete cosine transforms for the most and least merged female and male participants are shown. Changes in production over the course of the experiment can be seen in the data from all participants, and we examine the consistency and generalizability of those changes in analysis below.

The least merged female participant begins with distinct clouds for NEAR and SQUARE in her baseline productions (Pillai score = 0.89). The first shadowed productions are more merged overall (Pillai score = 0.65). However, the second shadowed productions are even more distinct than her baseline (Pillai score = 0.92), and she returns close to her baseline in the Post-task tokens (Pillai score = 0.80). The most merged female participant starts out with an almost completely overlapping distribution in her baseline productions (Pillai score = 0.07), and she remains fully merged throughout her shadowed productions (Pillai scores = 0.11, 0.00) and Post-task productions (Pillai score = 0.07). While her lexical sets do not become less merged, one can see in Figure 6 that the absolute position of the vowel cloud moves around the vowel space throughout the task; the distribution on the whole becomes more near-like in Shadowed 1, and moves to a higher F1 and lower F2 space in Shadowed 2 and the Post-task.

The least and most merged males are shown in Figure 7. The least merged male did not have distributions as distinct as the least merged female, showing separate, but adjacent clouds in his baseline productions (Pillai score = 0.76). The first shadowed productions show an overlap in categories (Pillai score = 0.42), but the second shadowed productions show almost distinct distributions (Pillai score = 0.78). Finally, in the post-task, his productions become even more distinct from one another (Pillai score = 0.85). The most merged male participant shows a pattern similar to the most merged female participant, with consistently merged productions across baseline (Pillai score = 0.01), Shadowed (Pillai scores = 0.18, 0.08), and Post-task productions (Pillai score = 0.06).

**Figure 8:**
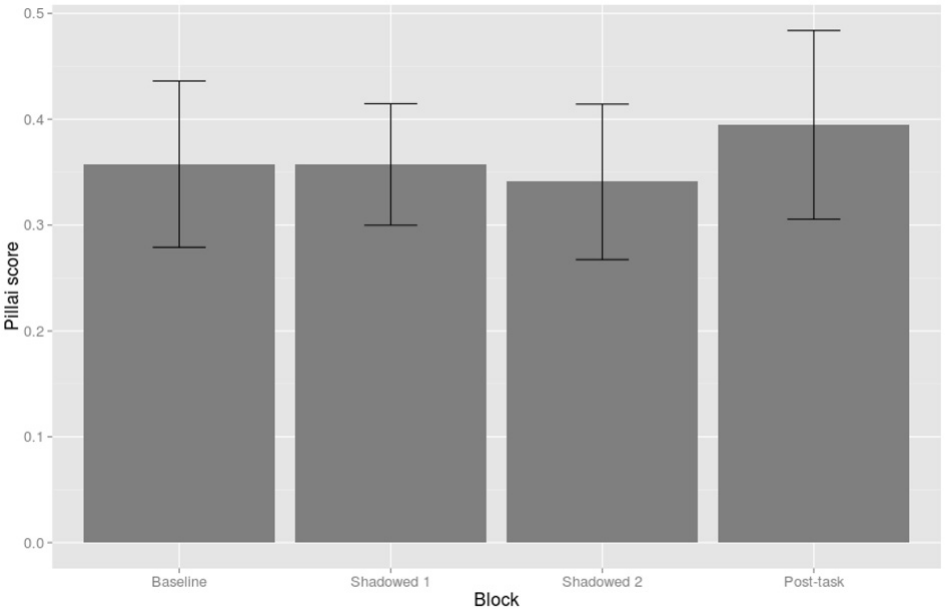
**Pillai score by Block.** Pillai scores of 1 represent wholly distinct distributions and 0 represent wholly merged distributions. Ninty-five percent confidence intervals are shown by error bars.

***By-Shadower linear mixed-effects model of Pillai scores.*** The linear mixed-effects model was constructed with Block (Baseline, Shadowed 1, Shadowed 2, and Post-task), Condition (Positive and Negative), and their interactions as fixed effects, and Shadower as a random effect with Block as a random slope. Block was treatment coded and, given that we were interested in changes in mergedness compared to baseline productions, baseline was set as the reference level. The overall intercept for the model was significant, as compared to 0 or fully merged, but it was closer to 0 than to 1 (β = 0.36, *SE* = 0.05, *t* = 6.72). The only significant effect in the model was that shadowers were less merged in the Post-task block compared to Baseline productions (β = 0.09, *SE* = 0.04, *t* = 2.27); this effect is shown in Figure 8.

**Figure 9:**
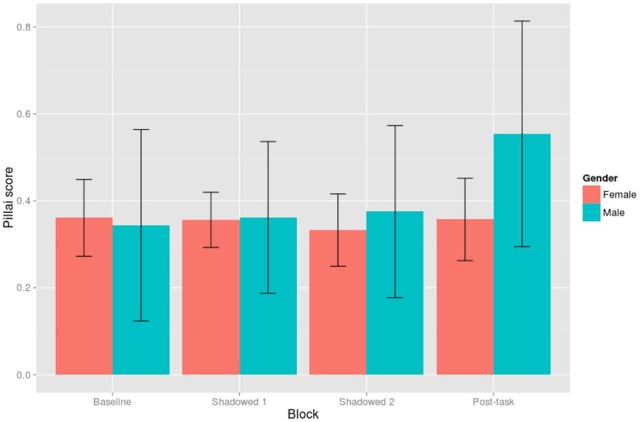
**Pillai score averaged by Block and Gender.** Pillai scores of 1 represent wholly distinct distributions and 0 represent wholly merged distributions. Ninty-five percent confidence intervals are shown by error bars.

Shadower gender was unbalanced in this study with only eight male shadowers compared to 34 female shadowers. In spite of this imbalance, *post-hoc* exploration of the data showed that male shadowers' speech behavior with respect to the merger differed from the female participants. As shown in Figure 9, we can see that the decrease in merger in the Post-task is an effect which is largely driven by male shadowers. This was shown by an interaction between Post-task and Male in a linear mixed effects model which was identical to the initial analysis but included Gender (β = 0.31, *SE* = 0.08, *t* = 3.72). The inclusion of Gender and its interactions in the model significantly improve its fit [χ^2^_(8)_ = 20.08, *p* = 0.01]. Note that in both Figures 8, 9 there is considerable variability amongst participants, as evidenced by the large error bars.

To parallel the analysis for listeners' judgments, we assessed whether shadowers' change in mergedness was correlated with their IAT score. The relationship was not significant.

**Figure 10:**
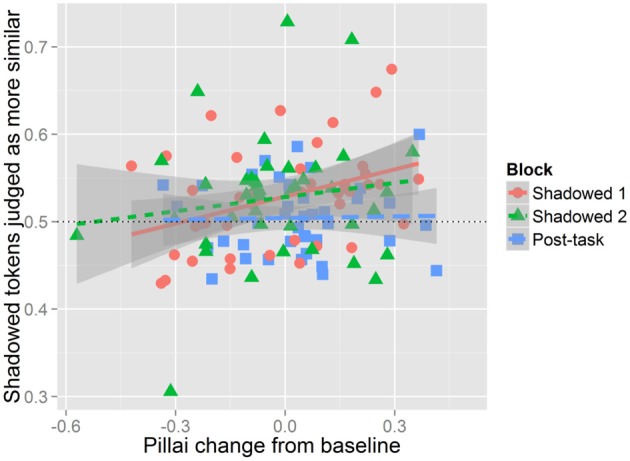
**Relationship between perceived accommodation and degree of mergedness.** Perceived accommodation is shown on the y-axis as the averaged proportion of Shadowed or Post-task tokens judged to be more similar to the model for each shadower in the Shadowed and Post-task blocks. Proportions above 0.5 indicate that Shadowed or Post-task tokens are more judged to be more similar to the model, while values below 0.5 means baseline tokens were more likely to be judged as similar to the model's production. Pillai change from baseline values are shown on the x-axis. A positive change value indicates a decrease in the degree of merger (i.e., becoming less merged), and a negative value indicates an increase in degree of merger (i.e., becoming more merged). A value of 0 would indicate no change in the degree of merger. Each dot on the figure represents the mean proportion of perceived convergence in a block for a single participant. Data from Shadowed 1 is in red, Shadowed 2 is green, and Post-task is blue.

***Listeners' judgments and mergedness.*** Shadowers' Pillai scores are calculated based on the distributions of their productions in each task block—Baseline, Shadowed 1, Shadowed 2, and Post-task. Given this, we have a single data point per block for each shadower. This precludes integrating the Pillai scores into the logistic mixed effects models reported in the above sections. Therefore, to assess the relationship between shadowers' mergedness and listeners' judgments of perceptual similarity we averaged the proportion of listeners' judgments indicating that the shadowed and post-task tokens sounded more similar to the model talker. This provides an individual measure of the relationship between changes in degree of merger and perceived accommodation independent of the group level patterns shown (or not shown) above. In other words, while the perceptual measure of accommodation found that as a group there was no accommodation in the Post-task and the acoustic measure found evidence for decreasing the degree of merger in the Post-task, individual differences in shadowers' production of the merger could still suggest that listeners used change in merger production as a cue for their similarity judgments. Indeed, this measure was modestly but significantly correlated with shadowers' change in mergedness across blocks compared to their baseline Pillai scores [*t*_(121)_ = 2.0, *r* = 0.18, *p* < 0.05]. This relationship is shown in Figure 10 which illustrates the positive relationship between listeners' judgments of accommodation and shadowers' changes in Pillai scores relative to baseline. Positive changes in Pillai scores indicate that shadowers are less merged, while negative values indicate an increase in mergedness. Listeners were more likely to perceive accommodation when shadowers' shadowed and post-task productions became less merged.

Crucially, this correlation does not indicate that listeners were exploiting the degree of merger as a cue toward phonetic imitation. Rather, it suggests that listeners *may* have made use this information in their judgments for the shadowing blocks, despite the finding that as a group, shadowers did not significantly decrease the degree of merger in the shadowing blocks. Figure 5 illustrates that *some* participants did decrease their degree of merger and that *some* of these participants were judged as having accommodated large amounts—these individual patterns were not seen across enough participants to be significant in the more comprehensive models reported above. Given the modest correlation, however, listeners were clearly using additional information beyond mergedness to assess perceptual similarity.

#### Discussion

Although participants did not immediately change their productions when faced with Australian productions, some participants did change their productions in the Post-task, becoming more unmerged and accommodating to the patterns of the Australian model talker.

Shadowers' change in degree of merger from baseline to their shadowed and post-task productions was correlated with listener' judgments of phonetic imitation. This finding means that it is possible that listeners were using the degree of merger as a way of assessing voice similarity. However, this result does not demonstrate that this *is* indeed what listeners were using when assessing the voices. The speech signal is rife with multidimensional information which listeners can use to determine similarity between voices. Beyond imitating spectral characteristics of vowels (Delvaux and Soquet, 2007; Babel, 2010, 2012), shadowers have been shown to accommodate to VOT (Shockley et al., 2004; Nielsen, 2011), fundamental frequency (Pardo, 2010; Babel and Bulatov, 2012), and duration (Pardo, 2010); the complex nature of the speech signal provides innumerable dimensions on which shadowers can accommodate.

## General discussion

This study examined spontaneous phonetic imitation with a merger-in-progress. Speakers of New Zealand English, a variety of English undergoing a merger of the NEAR and SQUARE lexical sets, completed an auditory naming task with a model talker who was a speaker of Australian English, an unmerged dialect with respect to this contrast. New Zealanders shadowed the tokens from the Australian model in either a Positive or a Negative Condition, which were intended to decrease social distance and increase social distance, respectively. After the shadowing task, participants took an Implicit Association Task to quantify their biases to New Zealand and Australia. We measured phonetic imitation in two ways: (1) we used a AXB perceptual similarity task which allows listeners to determine globally whether there was accommodation and (2) we quantified speakers' degree of merger using Pillai scores, and compared how the degree of merger changed for an individual from baseline to the Shadowed and Post-task productions. Our results indicate that these two measures do not lead to the same set of conclusions, and we integrate the two sets of findings in the paragraphs that follow.

Listeners' judgments indicated that shadowers accommodated more in the shadowing block than in their post-task productions. Generally, shadowers with pro-Australian IAT scores were judged as having accommodated: the more positively shadowers perceived Australia, the more they imitated, regardless of whether they were in the Positive or Negative Condition. This result generally replicates the association between phonetic imitation and IAT in Babel (2010) with the same set of speakers. Using only acoustic measures of imitation with a set of monophthongal vowels, Babel found that regardless of condition assignment, those with pro-Australian IAT scores were more likely to imitate and persist in that imitation. These IAT results, however, should be interpreted with caution (Blanton et al., 2009) and we acknowledge that while our IAT was intended to measure preference for Australia vs. New Zealand, it could well have been measuring amount of previous exposure to Australia or awareness about Australia or Australian English. That being said, IAT scores did correlate with perceived accommodation in the expected way: positive biases toward Australia predicted phonetic imitation. So, regardless of what the task actually measured, it accounted for some variability in speech production.

Listeners generally judged SQUARE words as having been imitated more than NEAR words. Listeners perceived this pattern for shadowers in the Positive Condition in the first and second shadowing blocks, and this pattern also emerged in the second shadowing block for those in the Negative Condition. Recall that our acoustic analysis assesses the degree of merger, not whether NEAR or SQUARE contributes to the shift more than the other lexical set. We see two possible scenarios for why listeners judged the SQUARE words as having been imitated more despite no global changes in mergedness in the shadowing blocks. Given that the merger is typically manifested as SQUARE shifting up toward the NEAR category, New Zealanders could have a larger phonetic repertoire for SQUARE words, allowing for more imitation with SQUARE (Babel, 2010; Kim et al., 2011) which was not captured in our analysis. A second scenario for why listeners judged SQUARE words as having been imitated more relates to what makes subtle phonetic changes perceivable for a listener. Walters et al. (2013) examined how voice similarity affects judgments of phonetic imitation. Listeners rated the similarity of shadowers' baseline productions to productions of the same word by model talkers on a visual analogue scale (Massaro and Cohen, 1983). A separate group of listeners completed an AXB perceptual similarity task to assess the degree of phonetic imitation. A comparison of the two sets of results found that for female voices there was a strong negative correlation between voice similarity and perceived accommodation: the more dissimilar a shadower's voice was to the model, the more listeners perceived phonetic imitation. This suggests that listeners may have an easier time assessing small phonetic changes along any dimension in a voice when that is more different compared to the model. Here, New Zealanders' productions of SQUARE words differ more from those of Australians than do the New Zealanders' productions of NEAR words. It is not possible to tease apart these two different interpretations for why listeners judged more imitation with SQUARE words based on the current data. But, the analysis of the Pillai scores does provide some clues. The analysis of the Pillai scores only found clear lessening of the merger in the post-task, most robustly for the small group of male shadowers, while in the perceptual analysis, listeners judged more imitation for the SQUARE words in the shadowing blocks for the Positive Condition and more in Shadowed 2 in the Negative Condition. There was no indication of parallel results in the Pillai scores, which suggests that listeners' abilities to perceive subtle acoustic changes on whatever diverse array of acoustic parameters shadowers may have been accommodating to is heightened when dealing with more different tokens for comparison. Moreover, we find that listeners' judgments of phonetic imitation are correlated with the amount which shadowers' mergedness changes from their baseline merger; the more shadowers decrease their degree of merger, the more listeners perceive accommodation.

Generally, with respect to the change in degree of merger, the analysis of Pillai scores revealed a decrease in merger in the post-task compared to baseline productions. In a *post-hoc* exploration of the data, we found that male participants were largely responsible for this pattern. On an individual level, however, shadowers did change their degree of merger throughout the task, but not in the most clear and predictable ways. Figures 6, 7, for example, illustrate how the least and most merged female and male participants shift the NEAR and SQUARE categories around their vowel space during the task. The least merged female (first row of Figure 6) exhibits her most clear cut categories in Shadowed 2, and the least merged male (first row of Figure 7) also clearly decreases his merger throughout the task. The most merged speakers, particularly the most merged female (second row of Figure 5), are shifting their phonetic categories throughout the task, but the entire NEAR/SQUARE cluster is shifting around together. This echoes the findings of Evans and Iverson (2007) who found that northern British speakers with merged *could* and *cud* items shifted the entire category toward Southern Standard British English *cud*, /Λ/, when immersed in SSBE.

## Conclusions

In this study we sought to examine whether individuals within a speech community undergoing a merger-in-progress can unmerge or decrease the degree of merger through spontaneous imitation in a task where the model talker was an unmerged speaker from a different dialect. The results suggest that speakers can change the degree of mergedness in such a task, but not necessarily in clear, incremental ways. We found considerable variability in how individuals merged and unmerged in the task, with the only clear group decreasing the degree of merger being the small number of males in the post-task block.

Listeners' assessments of phonetic imitation which extend beyond phonetic changes specific to the merger suggested that imitation increases with social preferences: New Zealander shadowers were more likely to accommodate if they had positive social biases toward Australia. These findings underscore the role of social or situational effects in the mapping of perception to production, and provide support for models of language in which speakers and listeners have access to phonetic representations with phonetic detail. The mismatch between listeners' perceptual judgments and the acoustic quantification of imitation suggests that phonetic imitation is a behavior which capitalizes on a rich array of phonetic information in the signal, and pin-pointing exactly *what* shadowers pick-up on in imitation can be somewhat of a wild goose chase.

### Conflict of interest statement

The authors declare that the research was conducted in the absence of any commercial or financial relationships that could be construed as a potential conflict of interest.

## Footnotes

^1^To clarify, with Shadowed 1 as the reference level for Block there was a three-way interaction with Post-task, Category (NEAR), and Condition (Negative). When the reference level for Block was Post-task, a three-way interaction with Shadowed 1, Category (NEAR), and Condition (Negative) surfaced. This indicates that the comparison between Shadowed 1 and Post-task with respect to Category and Condition is crucial.
